# Effects of Ultrasonic Treatment of Chicken Yolk on the Cryopreservation of Boar Semen

**DOI:** 10.3390/vetsci12111024

**Published:** 2025-10-22

**Authors:** Yanyan Liu, Fuqiang Chang, Biyu Zhang, Haidong Liu, Meng Zhou, Xin Zhang, Shouqian Sang, Xiu Li, Jing Li, Qianqian Hu, Youfang Gu, Chongmei Ruan

**Affiliations:** 1 College of Animal Science, Anhui Science and Technology University, Chuzhou 233100, China; liuyanyan20221229@163.com (Y.L.); changfuqiang2022@163.com (F.C.); 15255778563@163.com (B.Z.); 15256973237@163.com (H.L.); 13270885228@163.com (M.Z.); 18251925313@163.com (X.Z.); 19556959353@163.com (S.S.); lixiu@ahstu.edu.cn (X.L.); lij@ahstu.edu.cn (J.L.); 4713412@163.com (Q.H.); youfanggu@163.com (Y.G.); 2 Anhui Province Key Laboratory of Animal Nutrition Regulation and Health, Anhui Science and Technology University, Chuzhou 233100, China; 3 Anhui Engineering Research Center of Pork Quality Control and Enhancement, Anhui Science and Technology University, Chuzhou 233100, China

**Keywords:** ultrasonication, egg yolk, particle size, ROS, semen cryopreservation

## Abstract

**Simple Summary:**

Ultrasound treatment was applied to enhance the protective function of egg yolk in the cryopreservation of boar sperm. This study compared traditional and ultrasonicated egg yolk, finding that ultrasound enhanced yolk emulsification, leading to a finer, more stable emulsion. Sperm cryopreserved with the ultrasonicated yolk exhibited significantly better motility, higher membrane integrity, and increased post-thaw survival rates. Additionally, the activity of key antioxidant enzymes within sperm cells was elevated in the ultrasonicated yolk group. These findings demonstrate that ultrasound processing enhances the efficacy of egg yolk as a cryoprotective agent in boar semen preservation, suggesting potential benefits for swine breeding programs through improved frozen sperm quality.

**Abstract:**

Ultrasonic treatment significantly improves the emulsifying properties of chicken egg yolk. This advancement not only provides a novel approach for enhancing the physical stability of yolk-based cryodiluents, but also holds promising implications for optimizing the cryopreservation efficacy of boar semen. This study evaluated the effects of conventional egg yolk (CON) and ultrasonicated egg yolk (UT-CEY) on boar semen cryopreservation. Semen samples were cryopreserved using standard straw freezing methods, with post-thaw sperm quality parameters assessed. Results demonstrated that UT-CEY significantly reduced yolk particle size (*p* < 0.01), improved emulsion stability (*p* < 0.01), and decreased creaming index (*p* < 0.05). Additionally, UT-CEY enhanced total motility, progressive motility, straight-line velocity (VSL), and plasma membrane integrity (*p* < 0.01), along with acrosome integrity (*p* < 0.05) compared to CON. Furthermore, catalase (CAT) and superoxide dismutase (SOD) activities were elevated in UT-CEY (*p* < 0.01), while reactive oxygen species (ROS) fluorescence intensity showed no significant difference (*p* >0.05). Gene expression analysis revealed upregulated *Bcl-2*, *CAT* (*p* < 0.01), and *SOD_2_* (*p* < 0.05) in UT-CEY. In conclusion, ultrasonicated egg yolk diluent improves boar semen cryopreservation efficiency and post-thaw sperm quality.

## 1. Introduction

Semen cryopreservation technology plays a crucial role in minimizing disease transmission and overcoming geographical limitations, while also facilitating the long-term preservation of genetic resources. This technology is of critical importance for enhancing the utilization efficiency of elite breeding boars and reducing maintenance costs [1]. However, cryo-damage during freeze–thaw cycles—including ice crystal formation, osmotic stress, and oxidative injury—compromises sperm fertilizing capacity and may induce cell death [2]. Current research primarily focuses on optimizing freeze–thaw protocols and screening cryodiluent additives to mitigate sperm damage, thereby improving survival rates and fertilizing potential. Notably, porcine semen exhibits high seminal plasma ratios and low sperm membrane permeability, predisposing sperm to ice crystal formation during cryocooling, which induces cellular dehydration and structural disruption [3]. Furthermore, since porcine sperm membranes are enriched with polyunsaturated fatty acids (PUFAs), they are vulnerable to reactive oxygen species (ROS)-mediated lipid peroxidation (LPO) during freezing. This peroxidative damage compromises membrane integrity and functional stability, leading to sperm impairment or lethal injury [4]. Consequently, overcoming post-thaw quality deterioration caused by oxidative stress is pivotal for advancing large-scale application and commercial production of cryopreserved boar semen.

Egg yolk, serving as a non-penetrating cryoprotectant, is rich in phospholipids and lipoproteins. Its application in the cryopreservation of boar semen contributes to the stabilization of the sperm plasma membrane, thereby mitigating the deleterious effects of freezing damage and cold shock induced by ice crystal formation [5]. Furthermore, the cost-effectiveness and widespread availability of egg yolk render yolk-based extenders prevalent in practical applications. Nonetheless, the intricate protein composition of egg yolk, along with its substantial content of macromolecules and yolk granules, results in increased viscosity of the extender. This heightened viscosity not only impedes sperm motility but also complicates the microscopic evaluation of sperm. Ultrasound treatment, through mechanical agitation, cavitation, and microjets, can alter the spatial conformation of proteins and promote their interactions with small molecules [6]. Ultrasound treatment disaggregated chicken egg yolk granules, markedly reducing their average particle size, enhancing the solubility of yolk components, and thereby improving the emulsifying activity index [7]. Moreover, it effectively decreased emulsion viscosity, narrowed droplet size distribution, and significantly enhanced solution stability [8]. These changes collectively modify the functional properties of egg yolk and provide new insights into its application in boar semen cryopreservation. Nevertheless, few studies have reported the effects of ultrasound-treated egg yolk on boar sperm cryopreservation. Therefore, in this study, ultrasound-treated chicken egg yolk extender was applied to boar semen freezing, aiming to provide both theoretical basis and technical reference for optimizing boar semen cryopreservation extenders.

## 2. Materials and Methods

### 2.1. Experimental Animals and Management

Eight Large White boars aged 2–3 years, with strong libido, good body condition, and free from reproductive disorders, were selected from the Fengyang breeding farm in Anhui Province, China. All boars were individually housed in semi-open pens, provided with standard feed, and allowed free access to water. All experimental procedures were conducted in strict accordance with the guidelines approved by the Ethics Committee of Anhui Science and Technology University (Approval code: AHSTU2025004).

### 2.2. Reagents

All chemicals were analytical grade (Sigma-Aldrich, St. Louis, MO, USA) unless otherwise specified.

### 2.3. Solution Preparation and Processing

Fresh eggs were provided by Dingyuan Kangyuan Agri-animal Husbandry Technology Co., Ltd. (Chuzhou, Chian), and the cryodiluent for semen freezing was prepared according to the method described by HE et al. [9]. Coomassie Brilliant Blue staining solution was formulated by dissolving 0.2 g Coomassie Blue G-250 (Sangon Biotech, Shanghai, China) in 100 mL 95% ethanol, adding 200 mL 85% phosphoric acid, and adjusting to 2000 mL with distilled water before storage at 4 °C. Prepared diluents were divided into: conventional egg yolk (Control, CON) and ultrasonicated chicken egg yolk (UT-CEY) groups. CON diluent was homogenized using magnetic stirring (room temperature). All diluents were freshly prepared and stored at 4 °C prior to use. UT-CEY samples were treated by probe ultrasonication according to the method of Zheng et al. [10] with slight modifications. Briefly, samples were kept on ice during sonication at 150 W (Scientz-IID, Ningbo Scientz Biotechnology Co., Ltd., Ningbo, China) using a protocol of 5 s pulses followed by 20 s intervals, over a total processing time of 8 min.

#### 2.3.1. Particle Size Analysis and Microstructural Observation

Emulsion particle size distribution was determined using a laser diffraction particle size analyzer (Mastersizer 3000, Malvern Panalytical, Worcester, UK) following Liu et al. [11] with modifications. Measurements employed a dispersant refractive index of 1.330 and absorption index of 0.001, with dispersed droplet refractive index set at 1.456. All tests were conducted at room temperature and averaged over four replicates. Microstructure of diluents was examined via phase-contrast microscopy (EA211, Motic, Xiamen, China) with digital image acquisition.

#### 2.3.2. Emulsification Properties

Emulsification characteristics of ultrasonicated diluents were assessed according to Xie et al. [12] with modifications. Briefly, 50 μL emulsion was collected from the bottom phase, mixed with 5 mL 0.1% SDS solution, and absorbance at 500 nm was measured using a UV spectrophotometer (752N, Shanghai Yidian Analytical Instruments, Shanghai, China). Each sample was analyzed in quintuplicate. Emulsion stability index (ESI) and creaming index were calculated as follows.

$$EAI\frac{mL}{g}=\frac{4.606\times A_{0}\times N}{C\times F\times 10000}$$

$$\mathrm{creaming}\;\mathrm{index}\;\left(\%\right)=\frac{H_{c}}{H_{e}}\times 100$$

In the equations, A_0_ represents absorbance after homogenization; N denotes dilution factor (100); C indicates sample concentration (mg/mL); F signifies oil volume fraction in emulsion (0.25); H_c_ represents the height of the transparent liquid layer at the bottom (cm) and H_e_ represents the total height of the emulsion (cm). Each treatment was measured 5 times.

### 2.4. Semen Collection and Processing

Semen samples were collected using the gloved-hand method. Ejaculates were collected twice weekly from experimental boars, with a total of five collections per boar for sperm cryopreservation and quality assessment. Sperm motility was evaluated using a Computer-Assisted Sperm Analysis (CASA) system (Nanning Songjing Tianlun, Model ML-608JZ, Nanning, China). Only samples with total motility > 85% were selected and pooled for further analysis in this study.

### 2.5. Semen Cryopreservation and Thawing

**Figure 1:**
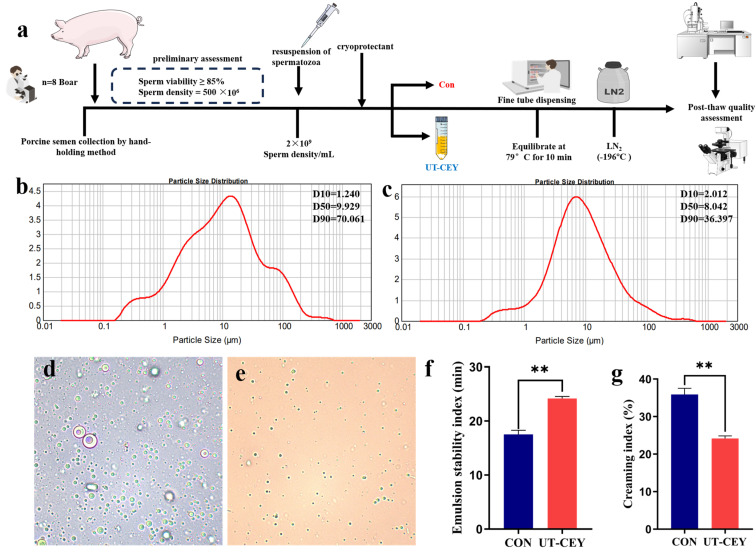
Effects of ultrasonic treatment on egg yolk particle size distribution, emulsion stability, and phase separation behavior. (**a**) schematic diagram of the semen cryopreservation process; (**b**) particle size analysis of CON mulsion; (**c**) particle size analysis of UT-CEY emulsion; (**d**) microstructural observation of CON extender; (**e**) microstructural observation of UT-CEY extender; (**f**) emulsion stability; (**g**) creaming index. ** denotes highly significant difference at *p* < 0.01.

The semen was diluted under aseptic conditions at 37 °C, with gentle mixing to minimize mechanical damage to spermatozoa. The diluted samples were subsequently equilibrated at 17 °C and then centrifuged at 800× *g* for 5 min prior to further experimentation. The diluted semen was centrifuged at 800× *g* for 5 min. The supernatant was then discarded, and the sperm pellet was resuspended in either conventional chicken egg yolk diluent or ultrasonicated chicken egg yolk diluen to achieve a sperm concentration of 2 × 10^9^ sperm/mL. Subsequently, the thoroughly mixed semen samples were dispensed into pre-labeled 0.5 mL plastic straws (Minitube, Tiefenbach, Germany) using a pipette and immediately sealed. The straws were frozen using the liquid nitrogen vapor method by exposing them to vapor 3 cm above the liquid nitrogen surface for 10 min before being plunged into liquid nitrogen for storage. For thawing, the frozen straws were removed from the liquid nitrogen tank and immediately immersed in a 50 °C water bath for 15 s [13]. The flowchart of the semen freezing process is shown in Figure 1a.

#### 2.5.1. Analysis of Sperm Motility Parameters

10 μL aliquot of thawed semen was placed on a pre-warmed (37 °C) Leja slide (ML-CASA, Mailang, Nanning, China) and analyzed using CASA configured according to Fraser et al. [14]. Sperm kinematic parameters were assessed based on the average of nine microscopic fields per sample [15].

#### 2.5.2. Assessment of Sperm Acrosome Integrity

Sperm acrosome integrity was evaluated using Coomassie Brilliant Blue staining according to Brum et al. [16] with minor modifications. Briefly, 200 μL of semen was centrifuged and washed, then resuspended and fixed in 1 mL of 4% paraformaldehyde for 8 min. After centrifugation at 2500× *g* for 3 min, the supernatant was discarded and the sperm pellet was resuspended. Smears were prepared using 10 μL of the suspension, air-dried, stained with Coomassie Brilliant Blue solution for 30 min, rinsed with water, and air-dried prior to examination. At least 200 spermatozoa were evaluated per sample to determine the rate of acrosome integrity.

#### 2.5.3. Plasma Membrane Integrity Rate

The integrity of the sperm plasma membrane was assessed using the hypo-osmotic swelling test (HOST). In a hypo-osmotic solution, viable spermatozoa with intact plasma membranes absorb water due to the osmotic gradient, resulting in tail curling or swelling. A 200 μL aliquot of semen was thoroughly mixed with 2 mL of hypo-osmotic solution and incubated in a 37 °C water bath for 1 h. A 10 μL sample was then placed on a glass slide and examined under a phase-contrast microscope at 400× magnification [17]. At least 200 spermatozoa were evaluated to determine the rate of acrosome integrity.

#### 2.5.4. Detection of MDA, SOD and CAT

The antioxidant status of thawed sperm was determined using commercial kits for malondialdehyde (MDA; Nanjing Jiancheng, A003-1, Nanjing, China), catalase (CAT; Nanjing Jiancheng, A007-1-1, Nanjing, China), and superoxide dismutase (SOD; Nanjing Jiancheng, A0013-3, Nanjing, China), according to the manufacturers’ instructions. The procedures were carried out strictly in accordance with the kit instructions. Colorimetric determination was performed using an enzyme reader (Thermo, Model 1510, San Diego, CA, USA), and the results were calculated according to the formulas provided in the kit instructions. Three replicate measurements were conducted for each sample group.

#### 2.5.5. Detection of ROS Levels

An appropriate volume of semen sample was processed according to the instructions provided with the Reactive Oxygen Species (ROS) Assay Kit (KeyGEN BioTECH, KGA7308-100, Nanjing, China). Briefly, the sample was centrifuged to remove the cryopreservation diluent, followed by the addition of the fluorescent probe working solution. Incubation was then carried out at 37 °C for 30 min in the dark. Subsequently, the sample was washed three times with PBS. Observation and image acquisition were performed using an upright fluorescence microscope (Olympus, BX63, Tokyo, Japan) under consistent exposure settings. For each sample, five randomly selected fields were captured. The average gray value of the fluorescence images was analyzed using ImageJ (ImageJ1.53a) software to evaluate the ROS levels.

#### 2.5.6. Detection of Apoptosis-Related Genes

Four frozen straws (0.5 mL each) were pooled and thoroughly mixed. The sample was washed three times with phosphate-buffered saline (PBS) for RNA extraction and subsequent real-time polymerase chain reaction (RT-PCR) analysis. Total RNA was isolated using the guanidine thiocyanate-phenol-chloroform method [18]. Reverse transcription was performed using the FastQuant RT Kit (Tiangen Biotech, Beijing, China). The resulting cDNA was diluted to 40 μL with nuclease-free water. Quantitative PCR was carried out using the SYBR Green IV fluorescence method (SYBR Green Premix Pro Taq HS qPCR Kit IV, Accurate Biotechnology, Changsha, China). cDNA sequences of apoptosis-related genes (*GAPDH*, *TNF-α*, *BCL-2*, *CAT*, *SOD_2_*) in boar sperm were retrieved from NCBI, and specific primers were designed. All primer sequences are listed in Table 1. Primers were synthesized by Sangon Biotech (Shanghai, China). GAPDH was used as the reference gene.

### 2.6. Statistical Analysis

Experimental data were analyzed using SPSS 26.0 software. The results are presented as the mean ± standard deviation (Mean ± SD). Comparisons between the two groups were performed using an independent samples *t*-test. A value of *p* < 0.05 was considered statistically significant.

## 3. Results

### 3.1. Effect of Ultrasonic Treatment on the Particle Size of Yolk Diluent

As shown in Figure 1, the mean particle size of the yolk diluent was determined using a laser particle size analyzer. The results indicated that 90% of the yolk particles in the UT-CEY were smaller than 70.061 µm (Figure 1b), whereas 90% of the yolk particles in the CON were smaller than 36.397 µm (Figure 1c). The particle size distribution of the ultrasonically treated yolk diluent was uniform, and the particle size was significantly reduced. Observations using an optical microscope revealed that the diameter of the yolk particles decreased after ultrasonic treatment, and the particles were smaller. In the diluent without ultrasonic treatment, the yolk particles formed a complex and irregular network structure with larger particle diameters (Figure 1d,e). The emulsion stability of the UT-CEY was significantly higher than that of the CON (*p* < 0.01) (Figure 1f), while the emulsion dilution index of the UT-CEY was significantly lower than that of the CON (*p* < 0.01) (Figure 1g).

### 3.2. Effect of Ultrasonicated Chicken Egg Yolk on the Kinematic Parameters of Post-Thaw Boar Sperm

As shown in Table 2, the effect of ultrasonicated chicken egg yolk diluent on the kinematic parameters of thawed boar sperm was evaluated. It was indicated that compared to CON, UT-CEY exhibited significantly higher values for Total Motility (TM), Progressive Motility PM, straight-line velocity (VSL), curvilinear velocity (VCL), and (average path velocity) VAP (*p* < 0.01).

### 3.3. Effect of Ultrasonicated Chicken Egg Yolk on Acrosome Integrity and Plasma Membrane Integrity of Post-Thaw Boar Sperm

**Figure 2:**
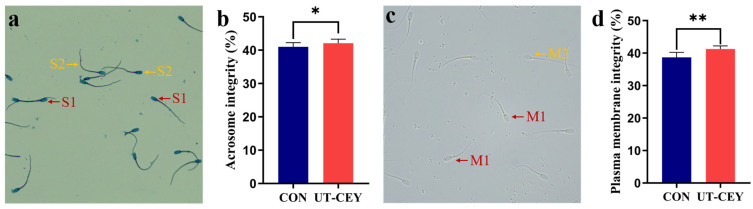
Effects of ultrasonic treatment of chicken egg yolk on acrosomal integrity and plasma membrane integrity of frozen-thawed boar sperm. (**a**) micrograph of acrosome morphology; (**b**) acrosome integrity (S1 denotes sperm with intact acrosome structure, S2 denotes sperm with damaged acrosome structure); (**c**) micrograph of plasma membrane morphology; (**d**) plasma membrane integrity (M1 denotes sperm with intact plasma membrane structure, M2 denotes sperm with damaged plasma membrane structure). * denotes significant difference at *p* < 0.05, ** denotes highly significant difference at *p* < 0.01.

As shown in Figure 2, the acrosome integrity rate was significantly higher in UT-CEY compared to CON (*p* < 0.05), and the plasma membrane integrity was also significantly greater in UT-CEY (*p* < 0.01). Sperm morphological examination revealed that spermatozoa with intact acrosomes exhibited a complete head structure and a distinct ridge line was visible, whereas those with damaged acrosomes showed obvious head defects and were more intensely stained (Figure 2a). A characteristic bending of the tail was observed in sperm with an intact plasma membrane (Figure 2c).

### 3.4. Effect of Ultrasonicated Egg Yolk on MDA, CAT, and SOD Levels in Post-Thaw Boar Sperm

**Figure 3:**
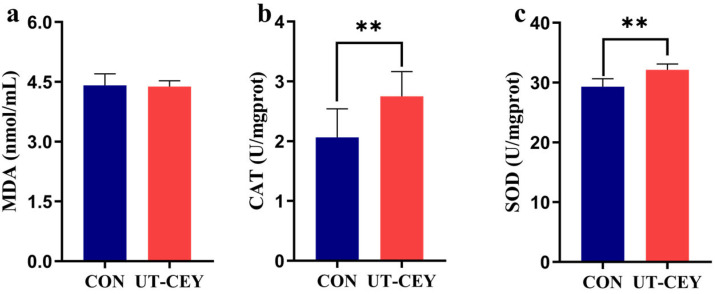
Effects of ultrasonicated egg yolk on oxidative stress parameters ((**a**) MDA, (**b**) CAT, (**c**) SOD) in post-thawed boar sperm. ** denotes highly significant difference at *p* < 0.01.

As shown in Figure 3, compared with CON, a reduction in MDA level was observed in UT-CEY (*p* > 0.05), while significant increases in CAT and SOD activities were recorded in UT-CEY (*p* < 0.01).

### 3.5. Effect of Ultrasonicated Chicken Egg Yolk on ROS Levels in Post-Thaw Boar Sperm

**Figure 4:**
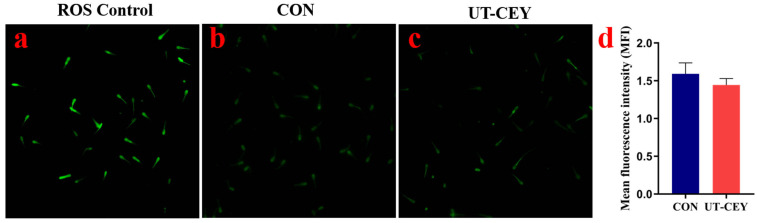
Effect of ultrasonicated chicken egg yolk on ROS levels in post-thaw boar sperm. (**a**–**c**) show ROS fluorescence images of the positive control group, CON, and UT-CEY, respectively; (**d**) shows the mean fluorescence intensity of ROS in sperm cells.

As shown in Figure 4, compared with CON, a reduction in ROS levels was observed in UT-CEY (*p* > 0.05). Fluorescence microscopy observations were consistent with these results.

### 3.6. Effect of Ultrasonic Treatment of Chicken Yolk on mRNA Expression Levels of Apoptosis-Related Genes in Boar Sperm After Freeze-Thawing

**Figure 5:**
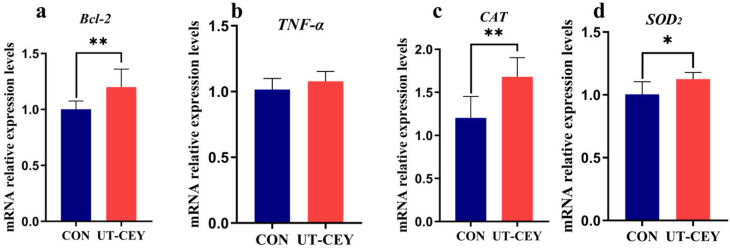
Effects of ultrasonic treatment of chicken egg yolk on expression of apoptosis-related genes in frozen-thawec boar sperm. (**a**) *Bcl-2*, (**b**) *TNF-α*, (**c**) *CAT*, (**d**) *SOD_2_*. * denotes significant difference at *p* < 0.05, ** denotes highly significant difference at *p* < 0.01.

As shown in Figure 5, compared to the CON, the expression level of *Bcl-2* was significantly increased in the UT-CEY (*p* < 0.01). The expression level of *TNF-α* was increased in the UT-CEY, but the difference was not statistically significant (*p* > 0.05). Compared to the CON, the expression level of CAT was significantly increased in the UT-CEY (*p* < 0.01). The expression level of *SOD_2_* was increased in the UT-CEY (*p* < 0.05).

## 4. Discussion

Ultrasonic treatment can alter the aggregated structure of yolk granules and significantly reduce their particle size through physical effects such as cavitation, mechanical shear forces, and microjets [19]. Studies hav e shown that ultrasonic treatment reduces the particle size of low-density lipoprotein (LDL) by approximately 50% and decreases solution turbidity, thereby further improving emulsion stability [20]. Ultrasonic treatment significantly enhances the emulsifying properties of wheat germ protein by not only increasing emulsifying activity and emulsion stability, but also effectively reducing the average droplet size and narrowing the size distribution. This improvement is attributed to enhanced protein adsorption at the oil-water interface and reduced interfacial tension, which collectively optimize the emulsification process [21]. Xu et al. [22] reported that ultrasonic treatment disrupts non-covalent bonds within protein or polysaccharide molecules, altering their conformation, unfolding molecular structures, and exposing more hydrophilic groups, thereby significantly improving solubility. Ultrasonic treatment optimizes yolk structure and emulsification properties, improving the performance of diluents and providing an effective new approach for optimizing semen preservation technology. Although ultrasonic technology has been widely applied in various fields, studies on ultrasonic treatment of yolk for boar sperm cryopreservation are scarce. Yolk is a common cryoprotectant in semen freezing protocols, where lipoproteins form a protective membrane that reduces cryodamage caused by ice crystals to the sperm membrane [23]. Yolk consists of 20% yolk granules and 80% yolk plasma. The addition of whole yolk during cryopreservation results in a turbid diluent with increased viscosity, which not only inhibits sperm motility but also interferes with sperm detection and analysis due to its opacity and residual large particles. Sperm motility parameters, which are core indicators of sperm movement performance and key factors in evaluating artificial insemination success, directly reflect sperm quality. Studies have shown a positive correlation between sperm motility, movement ability, and fertilization rates [24]. After high-intensity ultrasonic treatment, the diameter of yolk granules was reduced from 289.34 nm to 181.4 nm, and the contents of dry matter, protein, calcium, and phosphorus in the supernatant of the treated yolk solution were increased [7]. The results of this study demonstrated that sperm motility, viability, and straight-line velocity were higher in UT-CEY than in CON. This improvement may be attributed to the reduction in yolk granule diameter and enhanced dissolution of internal components following ultrasonic treatment, which improves diluent properties. Furthermore, sperm motility is positively correlated with the integrity of the cell membrane and acrosome. Therefore, the increased post-thaw sperm motility may be due to improved yolk emulsification and the protective effect of LDL on membrane integrity.

The plasma membrane, serving as the critical structural foundation for sperm to achieve fertilizing capacity, plays an indispensable regulatory role in fertilization processes such as sperm capacitation, the acrosome reaction, and sperm–egg recognition and binding [25]. Damage to the sperm acrosome or a premature acrosome reaction can lead to fertilization failure or abnormal embryonic development. The integrity of the plasma membrane is essential for maintaining normal exchange of substances and signal transduction between the intracellular and extracellular environments, effectively resisting oxidative damage, and ensuring sperm physiological function and fertilizing ability [26]. Phospholipids, cholesterol, and low-density lipoproteins (LDL) derived from egg yolk can incorporate into the sperm membrane, enhancing its stability and reducing cryo-induced damage caused by ice crystal formation and osmotic stress during freezing [27,28]. The results of this study indicated that both acrosome integrity and plasma membrane integrity were significantly improved in the UT-CEY compared to the CON. The enhanced cryoprotective effects observed with UT-CEY may be attributed to alterations in particle size and surface charge. These physicochemical properties not only influence the dynamics of interactions at the interface between particles and the lipid bilayer but also modulate their kinetic behavior at solid–liquid interfaces, thereby contributing to improved cryotolerance of sperm [29,30].

Spermatozoa possess an endogenous antioxidant system that scavenges free radicals during cryopreservation, thereby mitigating damage caused by oxidative stress [30]. However, boar sperm membranes are rich in polyunsaturated fatty acids (PUFAs), making them highly susceptible to oxidative stress during freezing [31]. Lipid peroxidation of PUFAs disrupts membrane fluidity, subsequently inducing sperm apoptosis and causing irreversible damage to membrane structure [32]. Therefore, reducing oxidative damage to sperm during cryopreservation is of particular importance. The results of this study demonstrated that higher enzymatic activities of CAT and SOD were observed in UT-CEY, indicating that the ultrasonically treated chicken egg yolk-based extender alleviated post-thaw oxidative stress in sperm. The antioxidant effects of egg yolk and its derivatives in sperm cryopreservation have been extensively documented. Through centrifugation processing and combination with other antioxidants, the cryoprotective efficacy of egg yolk has been optimized. These studies provide important foundations for improving sperm cryopreservation techniques and open new avenues for developing safer and more efficient cryoprotective agents.

When the sperm membrane is damaged by reactive oxygen species (ROS), its permeability undergoes irreversible disruption. This process can trigger the release of cytochrome c from mitochondria, subsequently activating apoptosis-related proteases such as caspase-9 and caspase-3, ultimately leading to sperm apoptosis [33]. The results of the present study demonstrated that the fluorescence intensity of ROS was reduced in UT-CEY, which may be attributed to the decreased particle size and improved emulsification activity of the treated egg yolk. The beneficial effect of egg yolk on sperm motility originates from its ability to elevate intracellular cAMP levels; its water-insoluble lipoprotein fraction contains factors capable of activating adenylate cyclase within the sperm plasma membrane [34]. Therefore, based on the aforementioned results, ultrasonic treatment contributes to reducing oxidative stress levels in sperm during cryopreservation by improving the physicochemical properties of egg yolk, thereby helping to maintain membrane structural stability, enhance antioxidant enzyme activity, and mitigate oxidative damage.

During cryopreservation, boar sperm experience oxidative stress, leading to elevated levels of ROS that adversely affect sperm quality and function [35]. Additionally, cryo-damage contributes significantly to sperm injury and death, with the extrinsic death receptor pathway playing a major role in inducing sperm apoptosis [36]. Apoptosis is an active, genetically regulated process of cell death that maintains cellular homeostasis [37]. As a pro-apoptotic gene, *TNF-α* can activate apoptotic signaling pathways, leading to impaired sperm function or even cell death. The expression level of *Bcl-2*, a key gene in the mitochondria-mediated apoptotic pathway, directly influences sperm survival. During cryopreservation, oxidative stress results in functional impairment and DNA damage in sperm. The activity of the antioxidant enzyme *CAT* is positively correlated with sperm motility. High expression of the *CAT* gene helps maintain mitochondrial function and ATP production, thereby supporting sperm motility [38]. Upregulation of *CAT* gene expression during freezing mitigates ROS accumulation—which can induce lipid peroxidation of the sperm membrane and DNA damage—thereby improving post-thaw sperm survival and fertilizing capacity [39]. The expression level of *SOD_2_* is positively correlated with the antioxidant capacity of sperm. Cryopreservation significantly reduces *SOD_2_* activity, leading to decreased mitochondrial membrane potential and reduced ATP production, which ultimately compromises sperm motility and fertilizing ability [40]. The results of this study revealed that the expression levels of both *CAT* and *SOD_2_* were elevated in the UT-CEY. This may be attributed to the improved physicochemical properties of the extender and enhanced antioxidant capacity, thereby alleviating oxidative damage to sperm during cryopreservation. Oxidative stress and apoptotic pathways play crucial roles in boar sperm cryopreservation, and the regulation of related genes is essential for maintaining sperm function. Although this study demonstrated that ultrasonic treatment of chicken egg yolk can significantly improve its physicochemical properties, thereby enhancing its cryoprotective effects and providing an efficient and cost-effective strategy for boar semen cryopreservation by improving sperm function and antioxidant capacity, the underlying molecular mechanisms remain incompletely elucidated. In particular, the interaction between apoptotic pathways and the antioxidant system requires further investigation. Future studies should focus on the precise modulation of key regulatory factors and integrate multi-omics technologies to optimize cryoprotective strategies, thereby not only improving the efficiency of semen cryopreservation but also facilitating its application in large-scale breeding and germplasm resource preservation.

## 5. Conclusions

Ultrasonic treatment effectively reduced the particle size of the chicken egg yolk-based extender and improved its emulsion stability and emulsifying activity index. In boar sperm cryopreservation, the treatment decreased ROS levels while increasing the rates of acrosome integrity and plasma membrane integrity. Furthermore, ultrasonic treatment significantly up-regulated the expression of genes associated with antioxidant and anti-apoptotic functions, such as *Bcl-2*, *CAT*, and *SOD_2_*. Overall, this study, for the first time, innovatively demonstrates the potential of ultrasonic-treated chicken egg yolk to enhance the cryoprotective effects of boar semen, providing a novel strategy for optimizing semen cryopreservation techniques and offering theoretical and technical guidance for commercial breeding and germplasm resource preservation.

## Figures and Tables

**Table 1:** qRT-PCR primer sequences.

Genes	Forward Sequence (5′-3′)	Reverse Sequence (5′-3’)	Product Length	NCBI ID
*GAPDH*	TTCCACGGCACAGTCAAGGC	CATGGTCGTGAAGACACCAG	150 bp	NM_001206359.1
*TNF-α*	ATTCAGGGATGTGTGGCCTG	CCAGATGTCCCAGGTTGCAT	120 bp	NM_214022.1
*Bcl-2*	GGCAACCCATCCTGGCACCT	AACTCATCGCCCGCCTCCCT	134 bp	NM_001322240.2
*CAT*	GCTGAGTCCGAAGTCGTCTA	GCCTTACAGAACGTTGCAGG	78 bp	NM_214301.2
*SOD_2_*	TTTGTAGGAGCGCCGAATAC	TAACCTCCTGGCTCTTTCCA	217 bp	NM_214127.2

**Table 2:** Effects of ultrasonically treated-chicken egg yolk diluent on motility parameters of frozen-thawed boar sperm.

Item	CON	UT-CEY	*p*-Value
TM (%)	31.43 ± 0.76 ^B^	35.00 ± 0.59 ^A^	0.000
PM (%)	25.60 ± 1.06 ^B^	30.25 ± 0.83 ^A^	0.000
VSL (μm/s)	20.59 ± 0.50 ^B^	21.32 ± 0.60 ^A^	0.004
VCL (μm/s)	35.22 ± 1.07 ^B^	36.93 ± 0.72 ^A^	0.000
VAP (μm/s)	28.68 ± 0.97 ^B^	31.20 ± 1.04 ^A^	0.000
LIN (%)	58.53 ± 2.31	57.74 ± 1.26	0.311
STR (%)	71.86 ± 2.43 ^A^	68.42 ± 3.06 ^B^	0.006
WOB (%)	81.48 ± 2.90 ^b^	84.49 ± 2.78 ^a^	0.016

Note: Different uppercase letters within the same row indicate extremely significant differences (*p* < 0.01), while different lowercase letters within the same row indicate significant differences (*p* < 0.05).

## Data Availability

The original contributions presented in this study are included in the article. Further inquiries can be directed to the corresponding author.
